# Direct Writing of Copper Micropatterns Using Near-Infrared Femtosecond Laser-Pulse-Induced Reduction of Glyoxylic Acid Copper Complex

**DOI:** 10.3390/mi10060401

**Published:** 2019-06-17

**Authors:** Mizue Mizoshiri, Keiko Aoyama, Akira Uetsuki, Tomoji Ohishi

**Affiliations:** 1 Department of Mechanical Engineering, Nagaoka University of Technology, Nagaoka 940-2188, Japan; 2 Department of Mechanical and Aerospace Engineering, Nagoya University, Nagoya 464-8603, Japan; pixwhxbl.color@gmail.com; 3 Department of Applied Chemistry, Shibaura Institute of Technology, Tokyo 135-8548, Japan; Mc18006@shibaura-it.ac.jp (A.U.); tooishi@sic.shibaura-it.ac.jp (T.O.)

**Keywords:** laser direct writing, femtosecond laser, glyoxylic acid Cu complex, reduction, Cu micropattern

## Abstract

We have fabricated Cu-based micropatterns in an ambient environment using femtosecond laser direct writing to reduce a glyoxylic acid Cu complex spin-coated onto a glass substrate. To do this, we scanned a train of focused femtosecond laser pulses over the complex film in air, following which the non-irradiated complex was removed by rinsing the substrates with ethanol. A minimum line width of 6.1 µm was obtained at a laser-pulse energy of 0.156 nJ and scanning speeds of 500 and 1000 µm/s. This line width is significantly smaller than that obtained in previous work using a CO_2_ laser. In addition, the lines are electrically conducting. However, the minimum resistivity of the line pattern was 2.43 × 10^−6^ Ω·m, which is ~10 times greater than that of the pattern formed using the CO_2_ laser. An X-ray diffraction analysis suggests that the balance between reduction and re-oxidation of the glyoxylic acid Cu complex determines the nature of the highly reduced Cu patterns in the ambient air.

## 1. Introduction

Laser direct writing of metal micropatterns has attracted attention from fields such as printed electronics and microelectromechanical systems. Two-dimensional (2D) metal micropatterns are generally fabricated using well-established methods of semiconductor technology consisting of lithography, metallic film deposition methods, and etching processes. However, deposition methods such as sputtering and evaporative coating must be done in an inert atmosphere, making it difficult to fabricate 2D metal micropatterns in air. In addition, multiple complicated steps such as lithography, metal deposition, and etching are needed to form metal micropatterns.

To overcome this problem, direct writing using laser-induced reduction has been proposed [1,2,3,4]. With this technology, Cu micropatterns are directly written using a laser-induced thermochemical reduction of copper oxide nanoparticles (NPs), such as CuO and Cu_2_O NPs, which are mixed with reductants and dispersants, and reduced to Cu by laser irradiation. When a CuO NP solution containing CuO NPs, polyvinylpyrrolidone (PVP), and ethylene glycol (EG) is irradiated by continuous-wave and nanosecond-pulsed lasers, acetaldehyde generated by dehydrating EG reduces the CuO NPs to Cu NPs, which are subsequently sintered to form Cu micropatterns [1]. When using a Cu_2_O NP solution, which contains Cu_2_O NPs, 2-propanol, and PVP, 2-propanol and PVP react thermally to generate formic acid, which then reduces Cu_2_O to Cu [2].

Two-dimensional Ni micropatterns can also be formed on glass and polyimide films using laser reductive sintering [3,4]. In this technique, NiO NPs mixed with toluene and α-terpineol are reduced to Ni by nanosecond-laser-induced thermochemical reduction. This technology has been used to fabricate Ni microwires with highly transparent electrodes on flexible films.

We have also fabricated Cu-based micropatterns using femtosecond-laser-reductive sintering of CuO NPs. An advantage of this approach is that the short pulse duration leads to rapid heating and cooling of the materials because the total irradiated energy can be reduced in femtosecond laser heating. In this research, irradiation by femtosecond laser pulses thermally reduces CuO NPs mixed with PVP and EG. Further, Cu- and Cu_2_O-rich micropatterns can be formed selectively by tuning the laser-irradiation conditions. The temperature coefficients of resistance of the Cu- and Cu_2_O-rich micropatterns are positive and negative, respectively, which is consistent with their respective metallic and semiconductive properties [5,6].

Metal complexes are promising candidate materials for direct laser writing using reduction, as demonstrated by the reduction of Cu complexes to produce 2D Cu micropatterns [7,8,9]. Typically, these Cu complexes are easily reduced at a relatively low temperature (~200 °C) [7]. In other work, Cu formate has been reduced to form Cu NPs by irradiation with an ultraviolet (UV) nanosecond-pulsed laser in an inert atmosphere under N_2_ gas flow [7,8]. A glyoxylic acid Cu (GACu) complex has also been developed for ambient-air Cu micropatterning using a CO_2_ laser [9]. This complex can be reduced in ambient air because of its high resistance to oxidation, ease of reduction, and strong absorption of CO_2_-laser irradiation. The minimum line width was ~200 µm, and the resistivity of the resulting Cu micropattern was ~3 × 10^−7^ Ω·m. However, finer line patterning has not been achieved because the line width depends on the irradiated diameter of the CO_2_ laser beam, which cannot be focused to a smaller spot diameter due to its long wavelength.

In this study, we report herein 2D Cu micropatterns fabricated in ambient air by using femtosecond laser reduction of a GACu complex to fabricate finer patterns with small line width. We first investigate the absorption properties of GACu, following which we discuss the patterning properties of GACu, such as resolution, crystal structure, and resistivity.

## 2. Experimental Methods

### 2.1. Direct Writing Process of Two-Dimensional Cu Micropatterns

**Figure 1:**
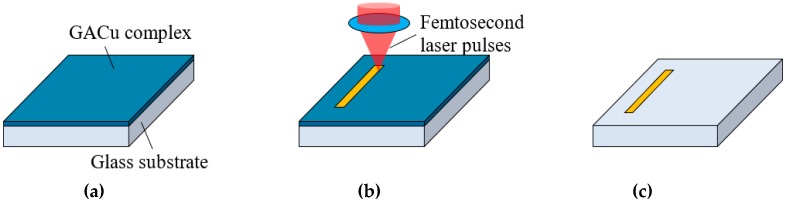
(**a**) Spin-coating of a glyoxylic acid Cu (GACu) complex film on a glass substrate. (**b**) Femtosecond-laser direct writing by reduction of the GACu complex film. (**c**) Nonirradiated GACu complex removed by rinsing the substrate with ethanol.

Figure 1 shows schematically the process for direct writing of 2D Cu micropatterns. A GACu complex was prepared using a previously reported method [9]. First, glyoxylic acid (4.5 mmol, Sigma Aldrich, St. Louis, MO, USA) dissolved in H_2_O (5 mL, FUJIFILM Wako Pure Chemical Corporation, Tokyo, Japan) was adjusted to pH 7 by adding NaOH aqueous solution (10 wt%, FUJIFILM Wako Pure Chemical Corporation). Next, CuSO_4_∙5H_2_O (4.5 mmol, FUJIFILM Wako Pure Chemical Corporation) dissolved in 5 mL H_2_O was added to the GA solution and stirred for three hours. The GACu complex precipitated from the solution and was filtered out, washed by H_2_O, and dried in a cooled, reduced-pressure atmosphere.

The GACu complex (6.0 mmol) was dissolved into a 2-amino-ethanol: ethanol (1:2, 3 mL, FUJIFILM Wako Pure Chemical Corporation) solution and was then spin-coated onto a glass substrate. The spin-coated film was heated at 50 °C using a hot plate for 30 min. To accomplish laser direct writing, we used a commercially-available femtosecond laser direct writing system (Photonic Professional GT, Nanoscribe GmbH, Eggenstein-Leopolds-hafen, Germany) to form Cu micropatterns by reducing and precipitating the GACu complex.

The wavelength, pulse duration, and repetition frequency of the femtosecond laser were 780 nm, 120 fs, and 80 MHz, respectively. The laser pulses had a Gaussian intensity distribution and were focused onto the surface of GACu complex films using an objective lens with a numerical aperture (NA) of 0.75. The focal spot diameter was 1.3 μm. The sample substrates coated with the GACu complex film were scanned using an xyz-piezo stage. The maximum scanning speed was 1000 µm/s.

### 2.2. Evaluation of GACu Complex Films and Cu Micropatterns

The absorption properties of the GACu complex film are important for laser direct writing. The absorbance of the film in the UV-to-visible range was examined using a UV-visible spectrometer (UV-2600 100V JP, Shimadzu, Kyoto, Japan). The line width was measured using field-emission scanning electron microscopy (FE-SEM). The crystal structure of the micropatterns was examined using X-ray diffraction (XRD) (Rint Rapid-S diffractometer, Rigaku, Tokyo, Japan). The diameter of the collimated X-ray beam was 0.3 mm, and the angle of incidence was 20°.

The resistance of the line patterns was measured using a multimeter (Truevolt series 34465A, Keysight Technology, Santa Rosa, CA, USA). The resistivity was calculated from the resistance and the cross section of the line patterns which were obtained using a surface coder (SURFCODER ET200, Kosaka Laboratory Ltd., Tokyo, Japan).

## 3. Results and Discussion

Here we discuss the properties of the Cu micropatterns on the SiO_2_ glass substrates. First, we examine the absorption of the GACu complex film, following which we investigate the properties of the micropatterns such as line width, the generation of Cu-based micropatterns, and their resistivities.

### 3.1. Absorption of the GACu Complex Film

**Figure 2:**
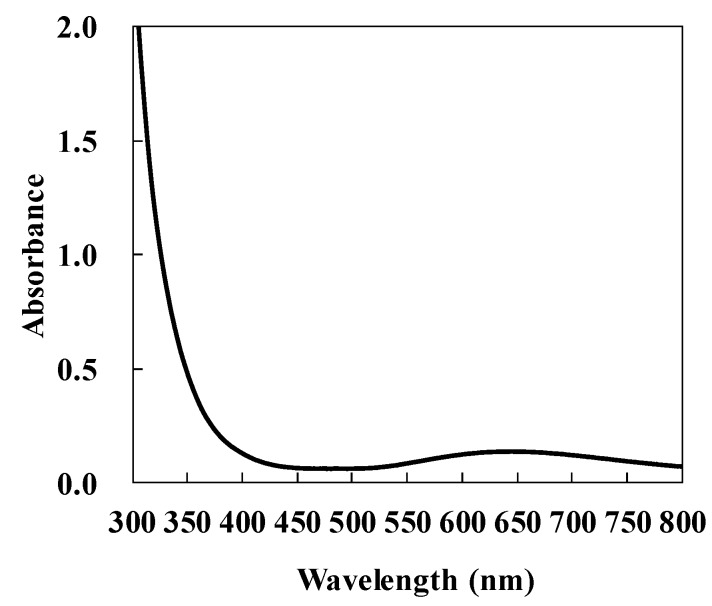
Absorption spectrum of GACu complex thin film.

Figure 2 shows the absorption spectrum of the GACu complex film on a glass substrate. The absorbance at 780 nm was almost the same as that at 390 nm, which indicates that single-photon absorption, rather than multi-photon absorption, is dominant during irradiation with femtosecond laser pulses at 780 nm. However, it is possible that three-photon absorption may occur. The relatively small absorption allows precise control of energy absorbed by the material by controlling the irradiated energy.

### 3.2. Patterning Properties

**Figure 3:**
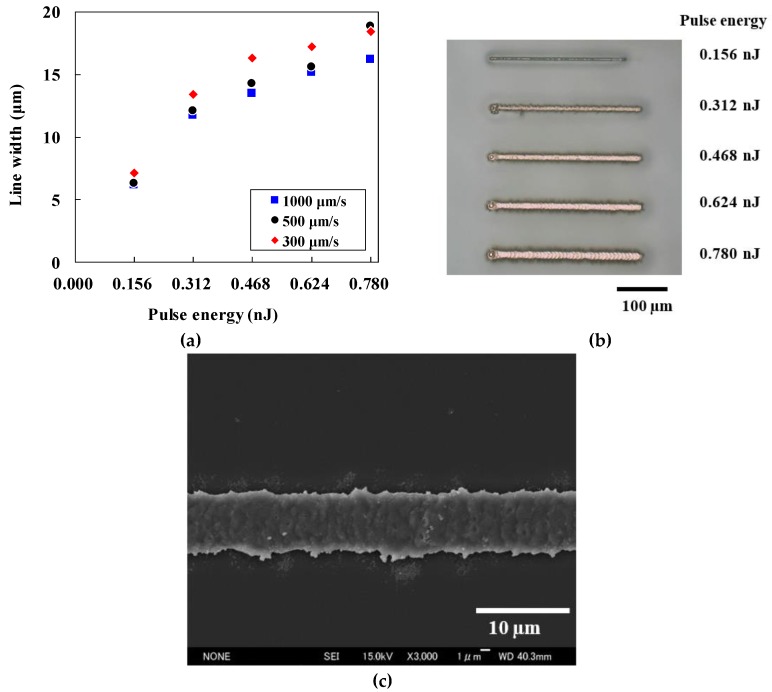
(**a**) Relationship between line width and laser irradiation conditions, (**b**) optical microscope image at scanning speed of 1000 µm/s and various pulse energies, and (**c**) field-emission scanning electron microscopy (FE-SEM) image showing the line width obtained when using a pulse energy of 0.156 nJ and scanning speed of 500 µm/s.

We next examined the relationship between pattern line width and laser-irradiation conditions, such as pulse energy and scanning speed. Figure 3a shows how the line width depends on the pulse energy at scanning speeds of 300, 500, and 1000 µm/s. Although scanning speed had relatively little effect on the line width, the width was affected significantly by the pulse energy.

An optical microscope image of the lines for evaluation is shown in Figure 3b. The scanning speed was 1000 µm/s and pulse energy was 0.156−0.780 nJ. We observed line patterns with a copper-like luster.

Figure 3c shows a FE-SEM image of a line pattern fabricated using a pulse energy of 0.156 nJ and a scanning speed of 500 µm/s. We observed the minimum line width of 6.1 µm at a pulse energy of 0.156 nJ and scanning speeds of 500 and 1000 µm/s; this is 7.4 times wider than the focal spot diameter. The greater line width appears to be due to the diffusion of thermal energy around the irradiated region. However, the line width is smaller than that previously obtained using CO_2_ laser reduction of a GACu complex [9] and using laser direct writing in air [1,5,7,8].

The direct writing of finer Cu wires is advantageous for the fabrication of integrated microdevices and of connections between electrodes. We expect that the line width can be reduced further by employing tightly focused femtosecond laser pulses using a high-NA objective lens.

### 3.3. Resistivities of the Line Patterns

**Figure 4:**
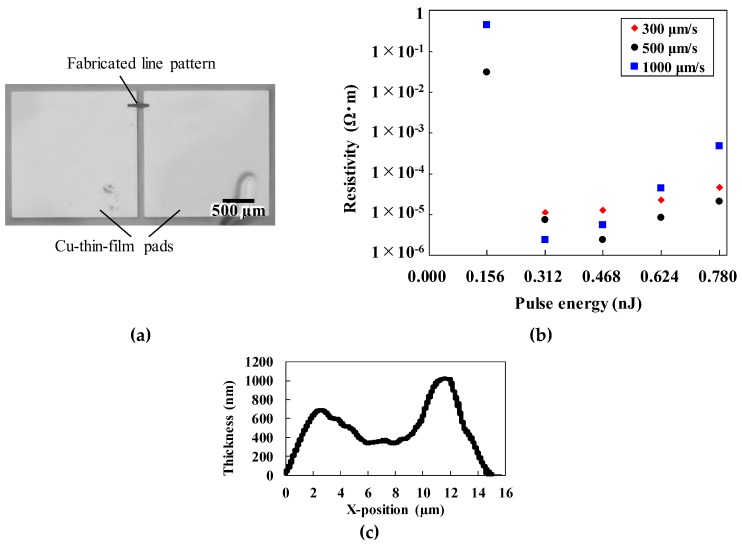
(**a**) Optical microscope image of a typical line pattern connecting two Cu thin film pads. (**b**) Resistivity of micropatterns fabricated under various laser irradiation conditions. (**c**) Cross-sectional profile of a line pattern produced at scanning speed of 500 µm/s and pulse energy of 0.468 nJ.

The resistivity of the line patterns was obtained by measuring the resistances and cross sections of the line patterns formed to connect Cu thin-film pads on a glass substrate. The size of each pad was 2 mm × 2 mm, and the gap between them was 110 µm which was the length of the line. The film thickness was ~300 µm. The resistance was less than 1 mΩ. Figure 4a shows a typical line pattern connecting the two Cu-thin-film pads, which were fabricated using lithography and sputtering methods. Figure 4b shows the resistivity as a function of pulse energy at various scanning speeds. Compared with the resistivities of the lines, the resistance of the Cu thin-film pads (<1 mΩ) is negligible. The minimum resistivity was 2.43 × 10^−6^ Ω·m for a line pattern formed with a pulse energy of 0.468 nJ and a scanning speed of 500 µm/s when the line width was ~14 µm as shown in Figure 3a. The line thickness was also estimated to be ~600 nm from the cross-sectional profile shown in Figure 4c. The center of the line was thinner than the sides. This indicates that the center was well sintered because of the higher central intensities of the laser pulses. This line width is significantly smaller than that obtained in the previous work, i.e., 200 µm [9]. However, the resistivity of the line pattern fabricated using femtosecond laser reduction was larger than the resistivity obtained in previous reports [9]. The resistivity increased at higher and lower pulse energies. The femtosecond laser pulse-induced rapid heating produced a combination of phenomena, such as the balance between reduction and reoxidation of Cu, sintering, and heat accumulation. The thermal history of the irradiated region must therefore be taken into account in order to determine the generated composition such as Cu and copper oxides. As a result, the line patterns are made of various composites of Cu and copper oxides under different laser-irradiation conditions.

### 3.4. Crystal Structures of the Micropatterns

**Figure 5:**
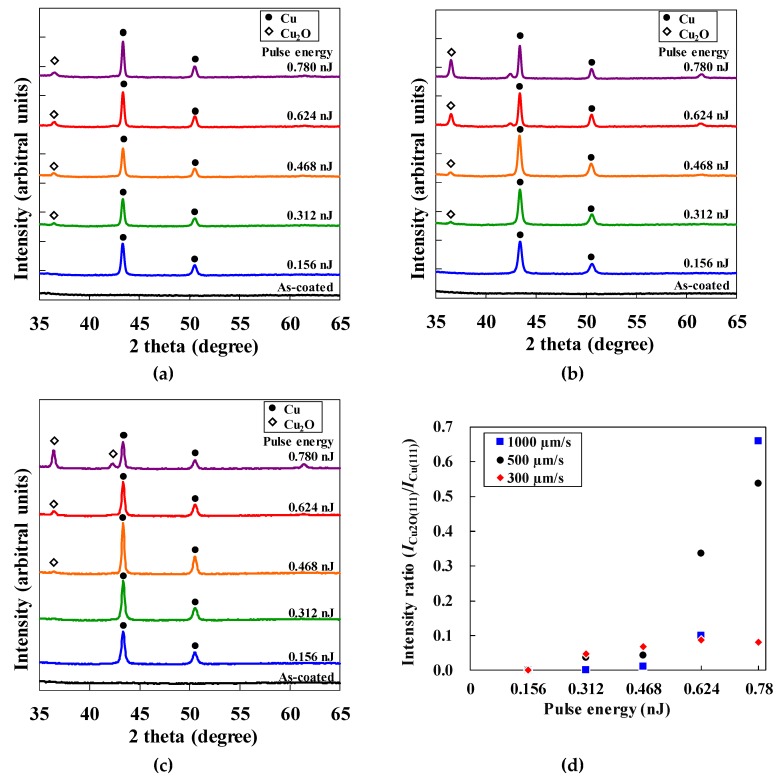
XRD spectra of fabricated micropatterns at a scanning speed of (**a**) 300 µm/s, (**b**) 500 µm/s, and (**c**) 1000 µm/s. (**d**) Intensity ratio of Cu_2_O to Cu as a function of pulse energy.

We now discuss the crystal structure of the micropatterns fabricated under various laser-irradiation conditions. The micropatterns measured 600 µm × 900 µm. The raster pitch of the micropattern was determined to be 1 µm by considering the laser focal spot of 1.3 µm. Figure 5a–c shows the XRD spectra of the micropatterns fabricated with scanning speeds of 300, 500, and 1000 µm/s, respectively. All spectra exhibit the diffraction peaks for Cu and Cu_2_O.

To compare the generation of Cu and Cu_2_O under different laser-irradiation conditions, we formed the XRD intensity ratio, for which the peak XRD intensity *I*_Cu2O(111)_ of Cu_2_O(111) was divided by that of Cu(111), *I*_Cu(111)_ (i.e., *I*_Cu2O(111)_/*I*_Cu(111)_). Figure 5d shows this intensity ratio as a function of pulse energy. The generation of Cu_2_O increases with increasing pulse energy for all scanning conditions, which indicates that Cu_2_O is generated by re-oxidation of previously generated Cu. The larger amount of Cu_2_O generated at high pulse energy is attributed to the grown Cu NPs generated by reduction at low scanning speed and that is difficult to re-oxidize, thereby preventing an increase in Cu_2_O.

By accounting for the generation of Cu and Cu_2_O, the increase in resistivity at high pulse energy is attributed to re-oxidation of previously generated Cu. In contrast, the increase in resistivity at low pulse energy suggests a lack of reduction of GACu. In general, the use of a short pulse duration prevents re-oxidation [10,11,12]. Controlling the temperature distribution and history in the line patterns may reduce the resistivity of the line patterns by changing the laser-pulse intensity distribution.

## 4. Conclusions

Cu-rich micropatterns were fabricated by femtosecond laser pulse-induced reduction of GACu complex.

(1) The minimum line width in the micropatterns was 6.1 µm, which was obtained with a laser-pulse energy of 0.156 nJ and scanning speeds of 500 and 1000 µm/s.
(2) The minimum resistivity of the line pattern was 2.43 × 10^−6^ Ω·m which was ~10 times greater than that of the pattern formed using a CO_2_ laser.

The results of the XRD analysis suggest that the balance of the reduction and the reoxidation of the GACu complex determines the ambient-air generation of highly reduced Cu patterns.
